# Shining new light on mammalian diving physiology using wearable near-infrared spectroscopy

**DOI:** 10.1371/journal.pbio.3000306

**Published:** 2019-06-18

**Authors:** J. Chris McKnight, Kimberley A. Bennett, Mathijs Bronkhorst, Debbie J. F. Russell, Steve Balfour, Ryan Milne, Matt Bivins, Simon E. W. Moss, Willy Colier, Ailsa J. Hall, Dave Thompson

**Affiliations:** 1 Sea Mammal Research Unit, Scottish Oceans Institute, University of St Andrews, St Andrews, Scotland; 2 Division of Science, School of Science Engineering and Technology, Abertay University, Dundee, Scotland; 3 Artinis Medical Systems BV, the Netherlands; 4 Sea Mammal Research Unit Instrumentation Group, Scottish Oceans Institute, University of St Andrews, St Andrews, Scotland; University of Oxford, UNITED KINGDOM

## Abstract

Investigation of marine mammal dive-by-dive blood distribution and oxygenation has been limited by a lack of noninvasive technology for use in freely diving animals. Here, we developed a noninvasive near-infrared spectroscopy (NIRS) device to measure relative changes in blood volume and haemoglobin oxygenation continuously in the blubber and brain of voluntarily diving harbour seals. Our results show that seals routinely exhibit preparatory peripheral vasoconstriction accompanied by increased cerebral blood volume approximately 15 s before submersion. These anticipatory adjustments confirm that blood redistribution in seals is under some degree of cognitive control that precedes the mammalian dive response. Seals also routinely increase cerebral oxygenation at a consistent time during each dive, despite a lack of access to ambient air. We suggest that this frequent and reproducible reoxygenation pattern, without access to ambient air, is underpinned by previously unrecognised changes in cerebral drainage. The ability to track blood volume and oxygenation in different tissues using NIRS will facilitate a more accurate understanding of physiological plasticity in diving animals in an increasingly disturbed and exploited environment.

## Abstract 2

A study using near-infrared spectroscopy in seals reveals that the mammalian dive response is not a reflexive response to facial immersion nor apnoea but may be under anticipatory cognitive control in voluntarily diving marine mammals.

## Introduction

The marked cardiovascular responses to submersion that diving mammals exhibit consist of bradycardia (reduction in heart rate [HR]), with decreased cardiac output (CO) and arterial constriction [1–19]. The resulting major redistribution of blood flow conserves oxygen by restricting perfusion of peripheral tissues nonessential to diving [4,10,13]. These physiological changes, collectively referred to as the ‘dive response’, have historically been assumed to be an autonomic response to submersion. Yet very little is known about the timing or magnitude of blood redistribution associated with the dive response in breath-hold diving animals, such as marine mammals, particularly at the level of individual tissues. Furthermore, given that cognitive control of HR has been demonstrated in diving mammals [6,7,13], it may also be that the onset of blood redistribution is not an entirely autonomic function. Real-time measurements of blood distribution from voluntarily diving animals may provide insight into whether there is any cognitive control over haemodynamics, as well as adding to existing information about diving oxygen management and physiological responses [14,15].

Much of our understanding of blood distribution and oxygenation during diving in marine mammals is based on haematological variables from the major vasculature using implanted intravascular oxygen sensors in freely diving pinnipeds [14,15]. However, information at the level of individual tissues is limited to cross-sectional measurements from lethally sampled animals after forced dives [4,10] and numerical modelling exercises [16]. From such data, it is known that marine mammals respond to submersion by reducing blood flow to the skin, blubber, and viscera [4,10], thereby delivering most of the CO to high-priority tissues such as the brain and, to some extent, the adrenals [4]. This redistribution of blood is achieved through peripheral arterial constriction with the consequent increase in peripheral resistance being compensated by a simultaneous and significant reduction in CO [11] (achieved by a rapid fall in HR, moderately reduced stroke volume, and reduced myocardial contractility [13]), matching left ventricular output to the restricted vascular beds and decreased venous return [17]. Therefore, the two classic features of the mammalian dive response—blood redistribution and bradycardia—are inextricably linked.

The onset of the dive response is elicited by medullary reflexes stimulated via peripheral tactile receptors and afferent nerves [13]. Thus, bradycardia is initiated at the onset of submersion, and minimum HR is established early in a dive [1,9]. The magnitude of bradycardia is greater in longer dives [1,9,12]. That the magnitude of bradycardia is both established early in a dive and is associated with dive duration indicates some aspect of cognition in the dive response. Indeed, cognitive control of HR has been suggested for numerous diving animals and demonstrated experimentally for both California sea lions (*Zalophus californianus*) [7] and harbour porpoises (*Phocoena phocoena*) [6]. Such cognitive influence on the dive response indicates impressive corticolimbic control of cardiovascular bulbar responses, enabling marine mammals to adjust their responses according to the anticipated challenge of the dive [13]. A few reports exist that indicate the level of corticohypothalmic influence on cardiovascular responses is so well developed that seals can elicit the dive response in anticipation of diving and/or surfacing [1,18,19]. How such cognitive control of the dive response affects blood distribution and oxygenation at the level of individual tissues is unknown; but given that arterial vasoconstriction is the primary component of oxygen conservation during diving, and not bradycardia [17] (albeit its secondary role is vital to avoid precipitous increases in arterial blood pressure), cognitive control of blood redistribution seems likely.

Despite major circulatory adjustments to reduce the rate of oxygen depletion, marine mammals can routinely experience extreme hypoxemia (low blood oxygenation), even during normal foraging dives [14,20]. The brain of seals seems well adapted to tolerating hypoxic exposure [21] through neurophysiological reconfiguration at the cellular and molecular levels [22,23], supported by vascular mediated cerebral hypothermia [24]. Nevertheless, blood redistribution and oxygen conservation associated with the gross circulatory changes that these animals exhibit are essential components of maintaining adequate cerebral oxygen delivery during diving [4]. Although muscle oxygenation measurements have been recorded from Weddell (*Leptonychotes weddellii*), harbour seals (*Phoca vitulina*), and emperor penguins (*Aptenodytes forsteri*) using surgically implanted near-infrared spectroscopy (NIRS) devices [25,26,27], the absence of practicable, noninvasive technology has limited our ability to gather information on blood volume and oxygenation from important organs and specifically the brain.

**Fig 1:**
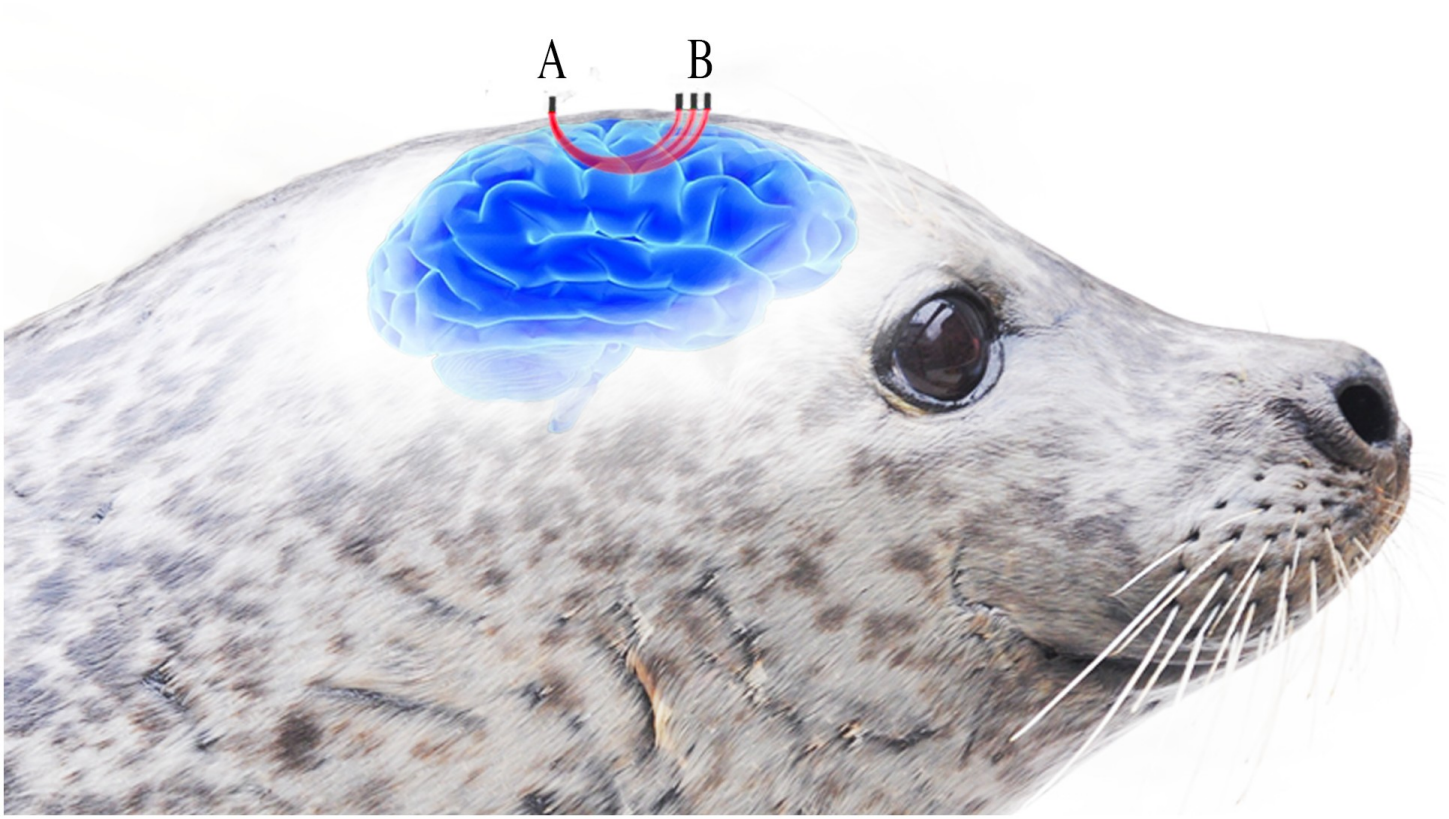
Visualisation illustrating the underlying concept of a three-optode-receiver-channel spatially resolved continuous wave near-infrared spectroscopy sensor. Heterodyning dual-wavelength light, visualised here in red, is emitted from (B) three light-emitting optodes in contact with a seal’s skin. Light passes through the underlying tissue before exiting the head, where it is detected by (A) a photodiode in contact with the seal’s skin. Increased distance between the optode and receiver channels provides deeper optical penetration within the underlying tissue.

Continuous wave (CW) spatially resolved spectroscopy (SRS) NIRS provides high-resolution relative measures of oxyhaemoglobin [ΔO_2_Hb] and deoxyhaemoglobin [ΔHHb] within specific tissues [28] (Fig 1). This allows calculation of total haemoglobin ([ΔtHb] = [ΔO_2_Hb] + [ΔHHb]) that can be used as a proxy for changes in blood volume and calculation of relative haemoglobin difference ([ΔHb_diff_] = [ΔO_2_Hb] − [ΔHHb]) that can be used as a proxy of oxygenation changes while removing the effect of changes in blood volume. Tissue saturation index (TSI), expressed as a percentage, provides a measure of the cumulative effects of changes in the concentration of total haemoglobin ([tHb]) and difference in the concentration of oxy- and deoxyhaemoglobin ([Hb_diff_]) on tissue oxygenation. Wearable NIRS systems developed for use in humans offer a first, minimally invasive system to investigate tissue oxygenation and blood volume in freely diving animals. We describe results from an application of NIRS technology (PortaLite mini, Artinis Medical Systems BV, Einsteinweg, the Netherlands) adapted for use on freely diving harbour seals (*P*. *vitulina*). We used a noninvasive, three-channel CW-SRS-NIRS system (Fig 1) to investigate the blood volume and oxygenation patterns in the brain and blubber during voluntary dives by captive harbour seals in a purpose-built diving foraging pool. NIRS data were obtained from four seals (two for cerebral measures and three for blubber measures) swimming freely in a quasi-natural foraging habitat. Detailed continuous records were obtained during hour-long sequences of 5.5 ± 1.08 min (mean ± standard deviation, unless otherwise stated) duration dives, typical of the behaviour of harbour seals in the wild [29]. We use the results to provide new insights into blood distribution and oxygen management in the blubber and brains of voluntarily diving seals and important information about basic physiological control mechanisms/functions associated with diving.

## Results

Synchronised behavioural and NIRS data were collected and analysed for 179 dive cycles (dive plus following surface period) across 27 experimental trials, from four animals (S1 Table). The seals performed a continuous series of dives (mean dive duration 5.54 ± 1.08 min) separated by short breathing bouts (mean duration 43 ± 8 s) in all trials. NIRS data were collected from blubber tissue and brain tissue for 102 and 77 of these dives, respectively. There are no synchronous blubber and brain measurements, as only one prototype NIRS device was available. NIRS measurements of [tHb], [Hb_diff_], and TSI showed repeated patterns, in both the brain and blubber, that were consistent between dives, across trials, and between animals. Although the magnitude of changes varied between dives, the overall dynamics of the observed changes remained consistent.

### Blubber blood volume and oxygenation dynamics

**Fig 2:**
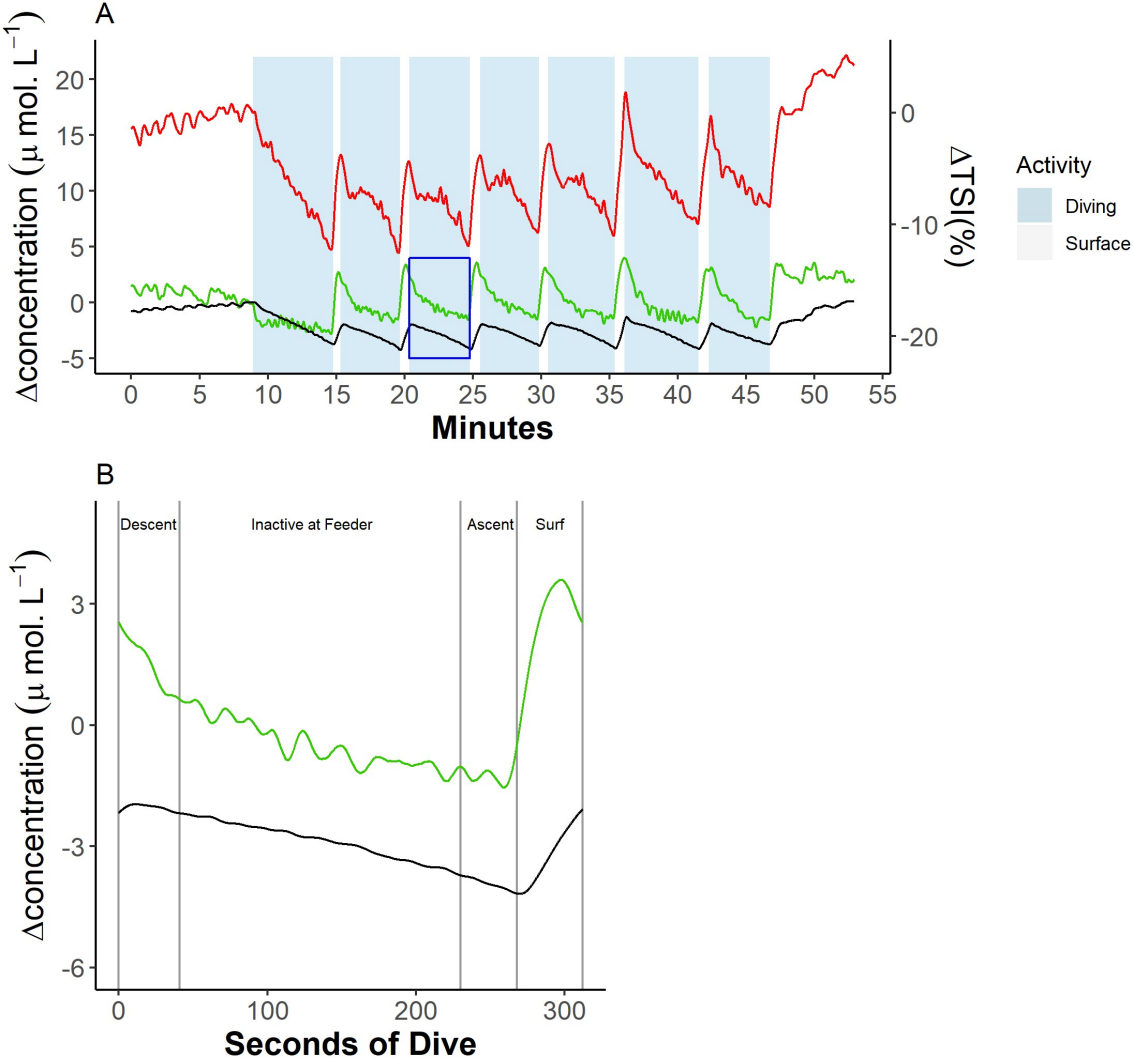
Example trace of blubber blood volume and haemoglobin oxygen dynamics during a sequence of dives from a single animal (Ulf). (A) Blood volume [tHb], haemoglobin oxygenation [Hb_diff_], and ΔTSI% dynamics across all the dives in a trial, where the green line represents blood volume, the black line represents haemoglobin oxygenation, and the red line represents TSI. (B) [tHb] and [Hb_diff_] dynamics across a single dive marked in blue in (A). Vertical grey lines indicate changes of dive phase, where ‘Descent’ = transit from the surface to the feeding station (feeder), ‘Ascent’ = transit from the feeding station back to the surface, and ‘Surf’ = at the surface. Data can be found here: https://doi.org/10.5061/dryad.k67cg66. [Hb_diff_], difference in the concentration of oxy- and deoxyhaemoglobin; [tHb], concentration of total haemoglobin; TSI, tissue saturation index.

**Fig 3:**
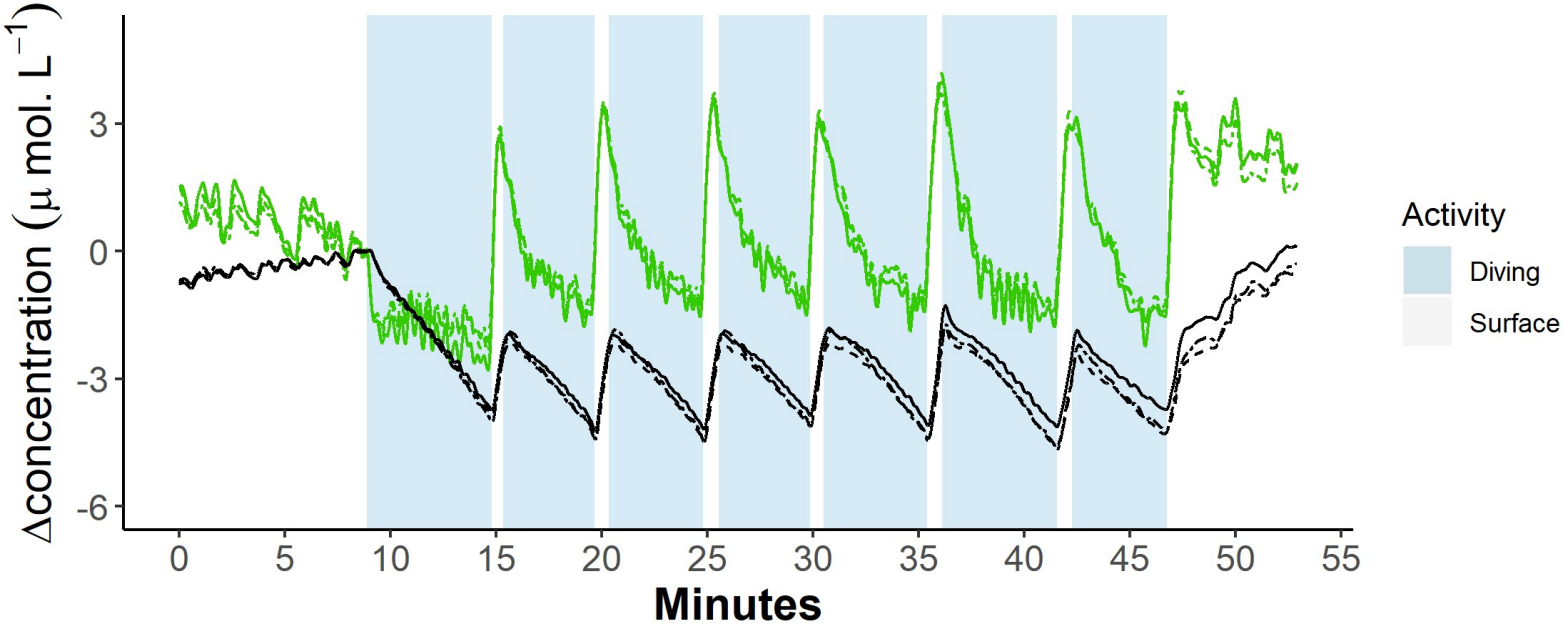
Example trace of blubber blood volume and haemoglobin oxygen dynamics from each of three optode channels during a sequence of dives. Green lines represent blood volume [tHb], and black lines represent haemoglobin oxygenation [Hb_diff_]. Dot and dashed lines represent measurements from the shallowest channel (28 mm), dashed lines represent measurements from the middle channel (33 mm), and solid lines represent measurements from the deepest channel (38 mm). Data can be found here: https://doi.org/10.5061/dryad.k67cg66. [Hb_diff_], difference in the concentration of oxy- and deoxyhaemoglobin; [tHb], concentration of total haemoglobin.

The dynamics of [tHb] and [Hb_diff_] showed a cyclic pattern over repeated dive cycles (Fig 2) that was common across animals (Fig 2; S1 Fig). The kinetics of both [tHb] and [Hb_diff_] showed similar patterns in each of the three optode and receiver separation distances (Fig 3; S1 Fig). During the 5–15 min period before the beginning of a diving trial, [tHb] and [Hb_diff_] were constant except for brief periodic reductions during short (<30 s) submersions of the head.

#### Diving

Following the onset of each dive, [tHb] declined rapidly. The date of decline in [tHb] slowed during the descent phase of a dive (Fig 2B). Rate of decline in [tHb] remained relatively constant throughout the remainder of the dive, reaching a minimum approximately 10 s before surfacing. [Hb_diff_] showed an initial increase for ≤10 s (±2 s) during the descent phase, reaching a maximum and TSI oxygenation maximum for each dive. [Hb_diff_] then declined monotonically throughout the remainder of the dive (Fig 2). Towards the end of a dive, about 10 s before surfacing, [tHb] increased rapidly, whereas the rate of [Hb_diff_] decline increased and [Hb_diff_] reached a minimum. [Hb_diff_] during diving remained below prediving levels.

#### Surface intervals

On surfacing, [tHb] continued to increase rapidly, reaching a maximum in the second half of the surface interval (Fig 2B). [Hb_diff_] and TSI increased steadily throughout this period. Maximum [tHb] during surface intervals was greater than prediving [tHb] levels, peaking at 62% ± 15% of the way through the surface interval. During the second half of a surface interval, [tHb] declined rapidly, whereas [Hb_diff_] and TSI continued to increase. Of the 102 dives, 30 did not display a reduction in [tHb] until after the start of the next dive. Of these 30 dives, 70% came from one animal—Thor (S1A Fig).

#### Postdive resting period

Following experiments, postdiving [tHb] remained higher than prediving levels throughout the resting period for at least 5–15 min. [Hb_diff_] did not reach prediving levels until 5–15 min of the postexperiment rest period.

### Cerebral blood volume and oxygenation dynamics

**Fig 4:**
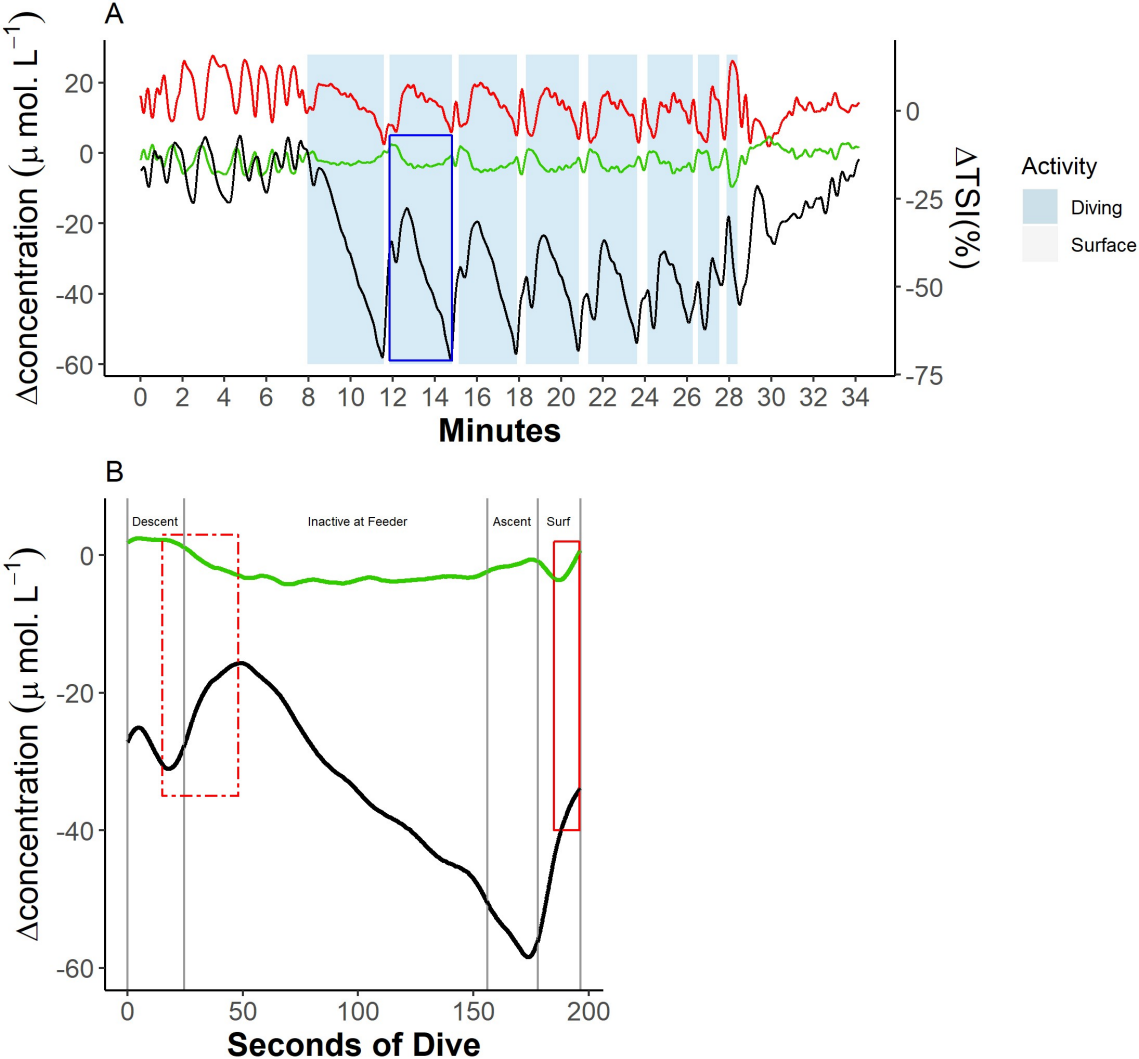
Example trace of cerebral blood volume and haemoglobin oxygen dynamics during a sequence of dives from a single animal (Ulf). (A) Blood volume [tHb], haemoglobin oxygenation [Hb_diff_], and ΔTSI% dynamics across all the dives in a trial, where the green line = blood volume, black line = haemoglobin oxygenation, and red line = TSI. (B) [tHb] and [Hb_diff_] dynamics across a single dive marked in blue box in (A). Vertical grey lines indicate changes of dive phase, where ‘Descent’ = transit from the surface to the feeding station (feeder), ‘Ascent’ = transit from the feeding station back to the surface, and ‘Surf’ = at the surface. The box in a dashed red line indicates a period of secondary reoxygenation. The box in a solid red line indicates a mid-surface interval increase in blood volume. All concentrations are expressed as a relative change from a baseline (0 μmol.L^−1^) taken as the onset of the first dive in a recording session. Data can be found here: https://doi.org/10.5061/dryad.k67cg66. [Hb_diff_], difference in the concentration of oxy- and deoxyhaemoglobin; [tHb], concentration of total haemoglobin; TSI, tissue saturation index.

**Fig 5:**
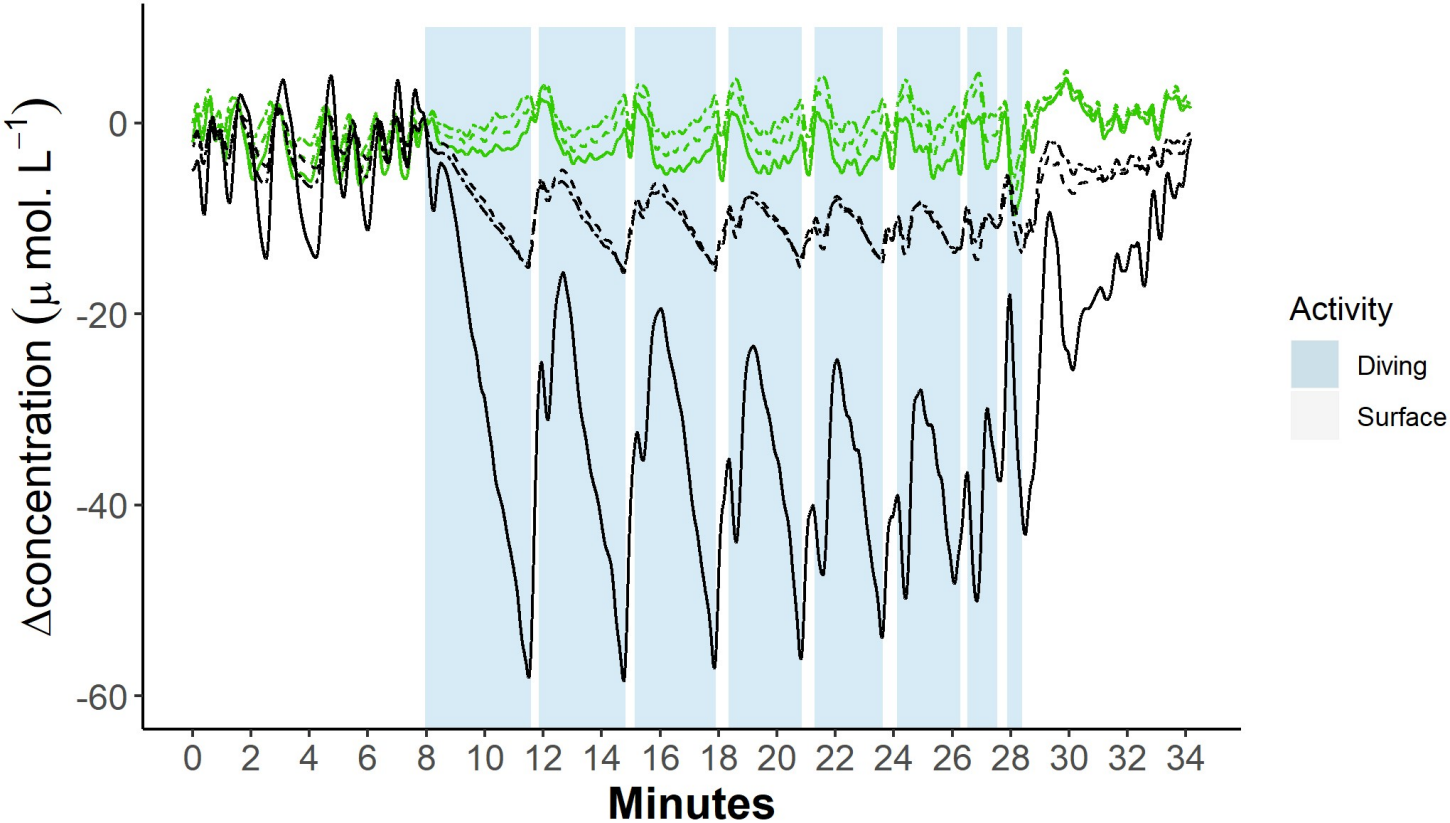
Example trace of cerebral blood volume and haemoglobin oxygen dynamics from each of three optode channels during a sequence of dives. Green lines represent blood volume [tHb], and black lines represent haemoglobin oxygenation [Hb_diff_]. Dot and dashed lines represent measurements from the shallowest channel (28 mm), dashed lines represent measurements from the middle channel (33 mm), and solid lines represent measurements from the deepest channel (38 mm). Data can be found here: https://doi.org/10.5061/dryad.k67cg66. [Hb_diff_], difference in the concentration of oxy- and deoxyhaemoglobin; [tHb], concentration of total haemoglobin.

The dynamics of [tHb] and [Hb_diff_] showed a cyclic pattern over repeated dive cycles (Fig 4) that was common across animals (Fig 4; S2 Fig). The kinetics of both [tHb] and [Hb_diff_] showed similar patterns in each of the three optode and receiver separation distances (Fig 5). There was, however, a noticeable increase in the magnitude of change in [Hb_diff_] in the deepest channel (38 mm) (Fig 5; S2 Fig). During the 5–15 min before the onset of diving trials, prediving [tHb] and [Hb_diff_] remained constant except for periodic reductions during short submersions of the head.

#### Diving

**Fig 6:**
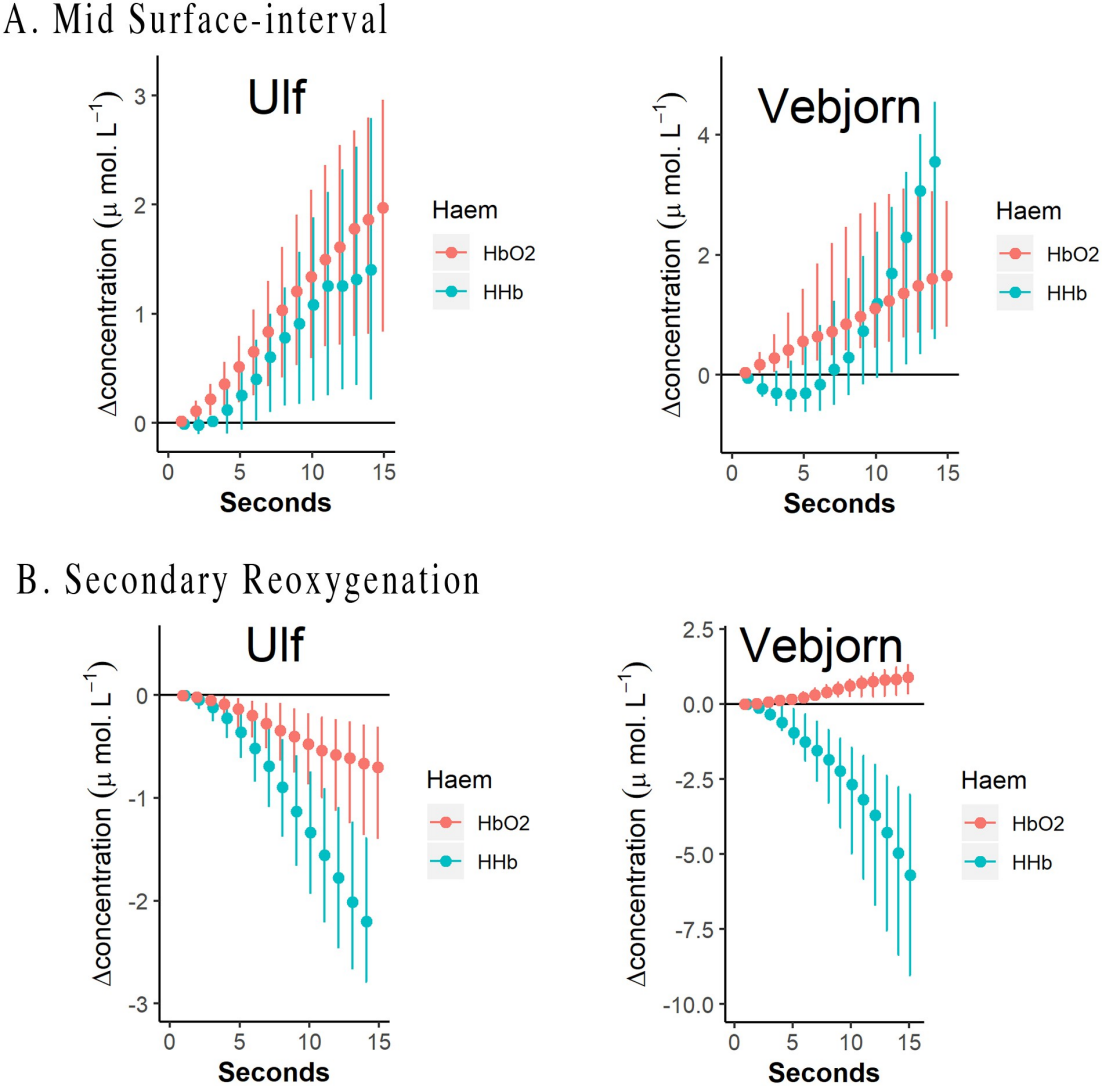
Cerebral haemodynamics (‘Haem’) of O_2_Hb and HHb for two seals during two key phases of a dive. (A) Mid-surface interval and (B) early in a dive when secondary reoxygenation occurred. Dots represent median concentration change. Error bars represent the interquartile range. Changes are shown as relative changes from CBV minima during a PDSI. Dives by Vebjörn, *n* = 23. Dives by Ulf, *n* = 54. Data can be found here: https://doi.org/10.5061/dryad.k67cg66. CBV, cerebral blood volume; HHb, deoxyhaemoglobin; O_2_Hb, oxyhaemoglobin; PDSI, postdive surface interval.

Following the onset of each dive, [tHb] increased, reaching a maximum 20 s (± 4 s) into the descent phase (Fig 4B). Throughout the period of increasing [tHb], [Hb_diff_] showed an initial increase during approximately the first 5 s of the descent phase, followed by a short rapid decline ending 20 s (± 4 s) into the descent phase (Fig 4B). Over this time course, TSI dropped rapidly. Following [tHb] maxima, [tHb] fell for the remainder of the descent phase and continued falling when the animals were inactive at the feeder (Fig 4B). This [tHb] decline was characterised by a greater reduction in [HHb] than in [O_2_Hb] (Fig 6B), and in one seal in fact [O_2_Hb] increased. As [tHb] declined, [Hb_diff_] showed a synchronous increase, forming a secondary peak (Fig 4B, dashed red line) approximately 50 s into each dive. Consequently, TSI increased rapidly throughout the period of secondary reoxygenation and falling [tHb]. On some dives, the secondary [Hb_diff_] peak represented a dive cycle [Hb_diff_] maximum—i.e., [Hb_diff_] during a dive was greater than at the beginning of the dive (Fig 4). After [tHb] minima and commensurate secondary [Hb_diff_] peak, [tHb] increased steadily while [Hb_diff_] and TSI declined steadily throughout the remainder of the dive until the animal surfaced. Cerebral [Hb_diff_] during diving remained below prediving levels.

In the diving trial shown in Figs 4 and 5, the last dive shows considerable, rapid reduction in [tHb] (Fig 4A), likely in response to human presence outside of the breathing chamber, which the seal did not expect. During the preceding surface interval, the seal appeared reluctant to surface, spending <5 s with the nostrils above the surface before crash diving and swimming rapidly away from the breathing chamber.

#### Surface intervals

On surfacing from a dive, cerebral [tHb] declined until after the midpoint of the surface interval (58% ± 0.2% of the surface interval) (Fig 4; S1 Fig). Conversely, [Hb_diff_] and TSI increased steadily throughout the first half of each interdive surface interval (Fig 4A). During some dive cycles, this fall in [tHb] during the postdive surface interval (PDSI) resulted in minimum [tHb] within a dive cycle. This pattern of reduction in cerebral [tHb] occurred in 71 of the 77 dives for cerebral NIRS measurements. Throughout the remainder of the surface interval, both [tHb] and [Hb_diff_] increased (Fig 4B, solid red box), whereas TSI decreased. Increased [tHb] was characterised by an initial increase in median [O_2_Hb], followed by a subsequent increase in [HHb] (Fig 6A).

#### Postdive resting period

Following experiments, postdiving [tHb] remained higher than prediving levels throughout the resting period for at least 5–15 min. [Hb_diff_] did not reach prediving levels until 5–15 min of the postexperiment rest period.

## Discussion

This study demonstrates the use of a noninvasive NIRS to investigate blood volume and oxygenation patterns in freely diving marine mammals. Patterns of blubber and brain blood volume and oxygenation shown here demonstrate that voluntarily diving seals make circulatory changes, as well as previously described changes in HR [6,7], in preparation for diving. Circulatory changes can occur well in advance of any changes in extrinsic stimuli and in advance of facial immersion and apnoea, when the dive response was thought to be initiated. The onset of the circulatory response, associated with classical diving responses, therefore appears to be under some cognitive control and is not a response stimulated by submersion itself. Furthermore, the NIRS results show an unexpected abrupt change in cerebral oxygenation partway through each surface interval and while submerged.

### Blubber blood volume and oxygenation dynamics

The reduction in blubber blood volume (BBV) prior to diving is consistent with haemodynamic changes associated with the onset of the dive response [4,8] and reflects a mechanism for both oxygen and energy conservation while submerged [4,5,13]. We argue this is an anticipatory response to diving because a reduction in blubber perfusion would serve little benefit in recovering from a previous dive but provides benefit to oxygen management in the following dive [4,13]. Eliciting peripheral vasoconstriction in advance of diving, thereby transitioning into a state of reduced oxygen consumption by minimising which tissues have access to circulating blood oxygen, could reduce the time during the initial phase of a dive when nonessential tissues are consuming blood oxygen stores that the dive response aims to conserve for high-priority tissues such as the brain [4]. Similarly, increasing peripheral restriction in advance of diving could facilitate the reduction in the partial pressure of oxygen in muscle tissue necessary to allow oxygen to be released from myoglobin, thereby supporting locomotor function with endogenous muscle oxygen stores rather than blood oxygen. Our data show a rapid increase in BBV, consistent with a fall in total peripheral restriction in anticipation of surfacing and coincident with the presurfacing tachycardia reported in free-ranging seals [1]—supporting the idea that reversal of the dive response is not reliant on the cessation of facial immersion or the onset of breathing [30]. Using NIRS, we have shown that initiation and termination of blood redistribution in phocid seals is therefore not elicited by apnoeic chemoreceptor stimulation (because changes occurred prior to breath hold as confirmed by video footage) or by peripheral tactile receptors [13]. Rather, preparatory changes in peripheral blood volume support previous work indicating cognitive control of HR in marine mammals [6,7] and show more extensive cognitive control of the cardiovascular system throughout a dive cycle than has previously been demonstrated.

Blubber [Hb_diff_] showed a monotonic decline throughout each dive (Fig 2). After an initial rapid drop in BBV, there was a gradual decline for the remainder of the dive (Fig 2), indicating that peripheral vasoconstriction occurs rapidly and then intensifies more slowly throughout each dive. Increased peripheral restriction facilitates delivery of oxygen-containing venous blood towards the heart and thus the brain [4].

BBV increased rapidly ≤ 10 s before surfacing, and this high perfusion rate continued throughout the first half of the surface period (Fig 2). These dynamics of blubber perfusion across dives were unexpected. As blubber is the primary source of insulation for phocid seals, and skin perfusion in homeotherms is ultimately dominated by the hypothalamic thermoregulatory centres, it was expected, teleologically, that blubber perfusion would be reduced through peripheral vasoconstriction [31] while seals were immersed in cold water. A consequence of repeated reperfusion of the blubber during surface intervals could be heat loss through the body surface. The thermoregulatory consequences would depend on whether a seal was attempting to conserve or dump heat. The dynamics of heat and blood flow regulation in the blubber are complicated. Venous plexi in seals are localised throughout the body but particularly in the blubber of the neck regions [32]. Blood flow through these plexi increases during diving [33]. Blix [5] suggested that venous blood draining from the head, which passes through the venous plexi in the neck while diving, seems to reduce body temperature. Furthermore, Blix and colleagues [34] have shown that diving seals can actively reduce body temperature, perhaps to slow metabolism and conserve oxygen. It is, therefore, possible that repeated peripheral perfusion between dives is a normal part of thermoregulation as well as oxygen management in seals. Rapid reperfusion of previously ischaemic tissues between dives will also help meet tissue oxygen demands and, at the level of the blubber, supply fatty acids to the circulation and facilitate removal of lactate, which likely accumulates during blubber ischaemia [35]. Anticipatory peripheral vasodilation may also ensure that lactate produced [35] can be recycled to pyruvate as soon as oxygen is available. Furthermore, peripheral vasodilation in anticipation of surfacing would facilitate CO_2_ removal from tissues and maximise CO_2_ concentration at the lungs at the start of the surface period. As CO_2_ elimination is slower than oxygen uptake [36], maximising CO_2_ elimination rates would minimise the time required for surface gas exchange.

### Cerebral blood volume and oxygenation dynamics

#### Changes in advance of diving

An increase in cerebral blood volume (CBV) occurred in advance of diving, representing a ≤3.2% increase in CBV (see S1 Text). The increase in CBV that occurred throughout the second half of each surface interval (beginning 58% ± 0.20% of the way through the surface interval) and the first 20 s of each dive (Fig 4) resulted from an increase in [O_2_Hb] and [HHb] over the first 15 s of this period of increasing CBV (Fig 6A) and thus in [tHb] (Fig 4). The net result of these increases was decreased TSI (Fig 4). Decreasing TSI, concomitantly with increasing [tHb], indicates a greater relative increase in [HHb] than [HbO_2_]. A greater relative increase in [HHb] is consistent with a mismatch between cerebral inflow and outflow, whereby the rate of inflow of blood into the brain exceeds the rate of outflow, and/or greater oxygen removal by cerebral tissue than can be met by delivery.

An increase in arterial pressure would explain the initial increase in cerebral [HbO_2_] in Fig 5B, as increased arterial pressure will increase cerebral blood flow and thus the delivery of oxygenated arterial blood to the brain [37]. If increased arterial delivery was matched by increased venous drainage, cerebral oxygenation would increase. However, if increased delivery of arterial blood is not matched with increased venous outflow, then cerebral oxygenation will decrease as the venous contribution of the intracerebral blood volume increases [37,38]. Increased central venous pressure inhibits venous outflow, thereby creating a mismatch in inflow and outflow from the brain, leading to pooling in the venocapillary system and decreased cerebral oxygenation [39–41]. We, therefore, hypothesise that decreasing BBV, which begins in advance of diving (and the likely onset of bradycardia), is indicative of increased peripheral restriction, which leads to increased arterial pressure (increasing cerebral blood flow to the brain). The resulting increased central venous pressure inhibits venous outflow, causing venous congestion, which can explain the greater relative increase in [HHb] as seen in Fig 6A. The consequences of increased arterial and central venous pressure would act synergistically to increase CBV and reduce cerebral oxygenation [37–43] and would explain why [tHb] increases yet TSI decreases during the second half of each interdive surface interval.

Seemingly concurrent with the increase of CBV during the second half of an interdive surface interval, BBV rapidly declined. A decrease in BBV in advance of diving is consistent with the onset of arterial restriction associated with the dive response [4], whereby blood flow is reduced to peripheral tissues nonessential to diving in order to conserve oxygen. As the onset of the reduction in BBV, in advance of diving, occurs 16.14 ± 8.00 s before diving, the onset of peripheral restriction appears to commence well in advance of diving bradycardia, which commences at the point of submersion [1,9]. Even if there was anticipatory bradycardia, which has been demonstrated in harbour seals [18], the onset of change in BBV in advance of diving in the current study almost always exceeded published durations of such anticipatory bradycardia (<2 s before diving). It would, therefore, follow from this reasoning that there is a period during the second half of each surface interval during which there is a mismatch between CO and arterial resistance. When CO exceeds the level of arterial resistance, arterial and venous pressures will elevate [13]. Indeed, in experiments in which CO has been artificially uncoupled from increased arterial sympathetic tone by inhibition of diving bradycardia (either by cardiac pacing [17], efferent vagal blockade [44], or atropine treatment [45]), arterial and venous pressures show a marked rise. At the level of individual tissues, such as the brain, increased arterial and venous pressures have the effect of both increasing blood volume and decreasing oxygenation [39–42], both of which were observed in the current study.

Decreasing cerebral oxygenation in advance of diving seems undesirable. However, if (as we hypothesise) the decreasing TSI is driven by intravascular pressure changes associated with the onset restriction, and cerebral deoxygenation can be tolerated, which it appears seals can [21–23], then decreasing cerebral TSI would allow seals to begin a dive in an already reduced oxygen consumption state. If this were correct, decreased TSI in advance of diving would not in itself be an anticipatory response but rather a physiological by-product of systemic perfusion changes.

An alternative explanation for the increase in CBV and reduction in TSI during the second half of each surface interval could be an increase in cerebral metabolic rate in which there is greater oxygen removal by cerebral tissue than can be met by delivery. This could explain both increased CBV and the relatively greater increase in [HHb] than [HbO_2_]. Increased cerebral metabolic rate, certainly during cortical activation, has an associated haemodynamic epiphenomenon in which there is arterial vasodilation and increased delivery of oxygenated blood. The result is that active cortical regions are characterised by increased [HbO_2_] and [tHb] but reduced [HHb] because the rate of blood oxygen delivery exceeds demand. This hemodynamic signal is the principal underpinning both blood oxygen level–dependent (BOLD) MRI and functional NIRS (fNIRS) bioimaging techniques [28]. We suggest, however, elevated cerebral metabolic rate is a less likely explanation for increased CBV in advance of diving than changes in intravascular pressure. First, the magnitudes of change in [tHb] during the hemodynamic epiphenomenon associated with increased cerebral metabolism are ≤1 μ.mol.L^−1^, and the changes in the present study are much greater (~5 μ.mol.L^−1^). Second, the epiphenomenon associated with elevated cerebral metabolic rate supports oxidative metabolism by ensuring that delivery of blood oxygen exceeds metabolic demand; hence, TSI increases. Yet, in the current study, TSI decreases, which is inconsistent with cerebral vasodilation associated with elevated cerebral metabolic rate. Admittedly, this pattern of reduced TSI could be a consequence of unique cerebral reconfiguration in seals [23]. The hypothesis of elevated cerebral metabolic rate in advance of diving and its consequence on blood volume and oxygenation could be tested using NIRS with inclusion of additional wavelengths to measure concentration of cytochrome c oxidase, which would capture changes in cerebral metabolic rate specifically and not just [HbO_2_] and [HHb] as in the present study.

#### Secondary reoxygenation

Seals abruptly reoxygenated cerebral tissue approximately 20 s into each dive, indicated by a marked increase in TSI (Fig 4A). During this ‘secondary reoxygenation’ phenomenon, declining [tHb] (Fig 4B) was dominated by a reduction in [HHb] (Fig 6B). Changes in intravascular pressure are again suggested here as the most probable explanation for the period of secondary reoxygenation. Following the onset of diving and bradycardia, it would be expected that adjustments in peripheral resistance, HR, and CO should allow arterial and central venous pressures to return to normal after the proposed increase during the latter part of the PSDI described above. Decreased central venous pressure should then promote increased venous outflow from the brain and thus reduce cerebral venous pooling [39–41]. Reduced venous pooling would simultaneously decrease CBV and increase TSI as a result of increased [HHb] removal. Decreasing the venous contribution of CBV would then increase cerebral TSI without any requirement for changes in systemic oxygenation. This explanation is consistent with the patterns here of decreased [tHb] early in each dive and increased TSI despite a lack of access to ambient air. Arterial–venous contributions within a tissue’s vascular bed are an important aspect of tissue-specific oxygenation in that tissue oxygenation relies not only on systemic blood oxygen levels but also on tissue-specific perfusion [40]. It is important to assess cerebral oxygenation directly rather than to infer oxygenation from systemic blood gas values [40].

While we do not have any blood pressure data to support our hypothesis for changes in arterial and/or venous pressures, we propose intravascular pressure changes as the explanation underpinning and ultimately linking the opposing patterns of change in CBV and cerebral TSI in advance of and shortly after each dive. Intravascular pressure measurements [46] and HR collected simultaneously with NIRS data would help support or refute our hypotheses.

An alternative explanation for the secondary reoxygenation phenomenon could be changes in systemic blood oxygen levels. The capacity for seals to infuse a bolus of oxygenated blood from the spleen into circulation during diving has been shown in freely diving Weddell seals and northern elephant seals (*Mirounga angustirostris*) [20,47]. A bolus of oxygenated blood resulted in an increase in haemoglobin (Hb) content in the major arterial vessels [20]. Contradictory responses of [tHb] and [Hb_diff_] in the current study suggests that a similar infusion of oxygenated blood from a central venous reservoir cannot fully explain the repeated pattern of cerebral oxygenation seen here. In the present study, there was a decrease in [tHb] in both the brain and blubber during secondary reoxygenation rather than increased [tHb], which could be expected if systemic Hb concentrations were elevated following infusion of previously sequestered blood. A lack of a secondary reoxygenation pattern in blubber also indicates the cerebral reoxygenation is not the product of systemic [HbO_2_] changes. Furthermore, injection of splenic stored blood cells is not a plausible explanation for the repeated reoxygenation across long sequences of dives. The short interdive surface intervals here (43 ± 8 s) make it unlikely that there would be sufficient time for seals to reoxygenate and replenish the splenic reservoir in time for repeated infusion across multiple dives. If a central venous reservoir is involved, it seems likely to comprise the hepatic sinus and caval vein [32].

A second alternative explanation for reduced CBV during the secondary reoxygenation phenomenon early in each dive could be a result of vasoconstriction of selected vascular beds in the brain. Blix and colleagues [4] showed reductions in brain blood perfusion in the early phase of the dive. Vasoconstriction would effectively reduce the cerebral vascular bed and thereby also reduce CBV. However, vasoconstriction would neither elevate TSI or [Hb_diff_] nor explain the greater relative reduction in [HHb] in the current study. We, therefore, suggest that changes in intravascular pressures are a more probable explanation for secondary reoxygenation accompanied by a simultaneous decrease in CBV.

#### Remainder of the dive

After the secondary peak in [Hb_diff_] and coincident [tHb] minimum for a dive, associated with secondary reoxygenation, CBV increased gradually throughout the remainder of a dive, whereas [Hb_diff_] showed a monotonic decline (Fig 4). Increasing CBV later in the dive may reflect localised hyperaemia in response to decreasing blood O_2_ and increasing CO_2_ [38] and demonstrates a similar perfusion pattern as shown by Blix and colleagues [4], as determined using microspheres. CBV declined rapidly on surfacing and throughout the first half of the surface period, consistent with rapid cerebral vasoconstriction, coincident with rapid cerebral reoxygenation (Fig 4) as seals reloaded depleted blood oxygen stores and off-loaded CO_2_ during postdive recovery. Alternatively, the decline of CBV on surfacing could be associated with the reduction in peripheral restriction [4].

As other authors have demonstrated, we show that seals do not fully reoxygenate Hb to prediving levels between repeated dives with short surface intervals, resulting in a downward trend in [Hb_diff_] over consecutive dives (Fig 4) [48]. Coincident with diminishing [Hb_diff_] at the start of successive dives, the magnitude of secondary oxygenation increased with each dive in a sequence (Fig 4). Increasing secondary reoxygenation in response to declining starting oxygenation could facilitate short surface intervals while maintaining adequate oxygen availability to the brain while submerged. The resulting shortened interdive surface intervals may allow seals to maximise time spent foraging and more fully exploit a transient prey patch while maintaining adequate oxygen delivery to key tissues that would otherwise be possible only if the seal remained at the surface until Hb was fully saturated.

Following the end of a diving bout, there was a protracted period of elevated [tHb]. Elevated [tHb] may be indicative of postexercise hyperaemia associated with recovery. Blix and colleagues [4] found similar increases in blood following diving and proposed it was a response to postdive recovery.

#### Limitations of method

Due to the novel application of NIRS in the current study, a number of assumptions had to be made about the optical properties of seal Hb and tissue. First, we have relied upon the fact that Hb of northern elephant seals has absorption peaks that are identical to cattle, sheep, and multiple other mammalian species, including humans [49], to assume that the properties of harbour seal Hb are also the same as human and elephant seal Hb. We argue that this is a reasonable assumption given the identical nature of Hb from a variety of mammals. Second, the absorption coefficient and reduced scatter coefficient for seal tissues are not yet published, and we therefore had to assume a differential pathlength factor (DPF) of six—equal to that used for human cerebral measurements as well as adult sheep [50]. Solving for the optical properties for seal tissues may affect the exact relative changes in micromolar units per litre of tissue for [HbO_2_] and [HHb] and therefore [tHb] and [Hb_diff_], too. A change in DPF of 1.0 will change the magnitude of change in [HbO_2_] and [HHb] by 20%, and a change in DPF of 2.0 changes the magnitude by 40% [51]. However, as the key focus of this manuscript is the patterns of change in tissue blood volume and oxygenation and the timing of these changes across repeated dive cycles, the conclusions and key results would be unaffected by a change in DPF.

### Conclusion

We present the use of a noninvasive waterproof NIRS system to measure continuous, high-resolution, dive-by-dive blood volume and oxygenation changes in the blubber and brain of voluntary diving seals. Our results provide insights into the mechanisms that underpin the exquisite oxygen management achieved by breath-hold divers. We clearly show repeated, highly reproducible patterns of changes in local blood volume and oxygenation that are indicative of cognitive control of circulation in diving seals and the likely use of increased venous drainage as a mechanism for modulation of cerebral reoxygenation during apnoea. These insights are a direct consequence of applying noninvasive technology that provides continuous measures of both blood volume and oxygenation. The success of NIRS in such an extreme environment highlights its potential for observing physiological processes in real-world contexts in the wild and in aquatic environments. The wearable and noninvasive nature of NIRS may offer a technology that could be integrated with existing animal-borne systems that measure behaviour and environmental metrics. NIRS could open new avenues for broad-scale physiological measurement synchronised with existing behavioural and environmental metrics used to assess responses of animals to environmental change and inform environmental policy.

## Materials and methods

### Ethics statement

Procedures for capture, handling, and housing of animals conformed to the Animals (Scientific Procedures) Act 1986, under the Sea Mammal Research Units’ (SMRU) Home Office licence (#70/7806).

### Experimental model and subject details

Diving trials were conducted with four temporarily captive harbour seals (*P*. *vitulina*) in a purpose-built foraging pool at the SMRU, St Andrews University [52]. All animals were wild caught from the Moray Firth, Scotland, and temporarily housed at the SMRU captive animal facility (University of St Andrews, Scotland) consisting of three unheated seawater pools. Procedures for capture, handling, and housing of animals conformed to the Animals (Scientific Procedures) Act 1986, under SMRU’s Home Office licence (#70/7806).

### Method details

#### NIRS system

An animal-borne archival NIRS sensor, PortaSeal, was developed from an existing wearable dual-wavelength CW-NIRS system for humans (PortaLite mini) that simultaneously uses the modified Beer-Lambert law [53] and SRS methods. Changes in the concentration of HbO_2_ and HHb can be calculated from changes in light absorption using a modification of the Beer-Lambert law, which describes optical absorption in a highly scattering medium [54]. Since the absolute concentration of chromophore is unknown, relative values for HbO_2_ and HHb were measured as a change from the start of the first dive following a 5–15 min prediving period in micromolar units per litre of tissue (μ.mol.L^−1^). This allowed calculation of total Hb ([ΔtHb] = [ΔO_2_Hb] + [ΔHHb]) that can be used as a proxy for changes in blood volume and calculation of relative Hb difference ([ΔHb_diff_] = [ΔO_2_Hb] − [ΔHHb]). Measures of [HbO_2_] and [HHb] allowed calculation of [tHb] and [Hb_diff_]. [tHb] provides information on Hb volume without the influence of relative oxygenation changes ([Hb_diff_]). [Hb_diff_] provides information on relative Hb oxygenation without the influence of changes in Hb volume. TSI is a measure of oxygenation (measured using a theoretical photon propagation model in a highly scattering medium [51,55]) that provides a measure of the collective effect [tHb] and [Hb_diff_] on tissue oxygenation. NIRS measures a volume of tissue containing microvasculature, comprising capillary, arteriolar, and venular beds but not arteries and veins. NIRS is weakly sensitive to blood vessels >1 mm in diameter because they completely absorb the light [28]. CW-NIRS has previously been used to measure [HbO_2_], [HHb], and [tHb] in nonhuman species such as sheep [50] and pigs [56] and TSI in species such as dogs [57]. Seal Hb has the same optical properties as human Hb [49], allowing the previous application of invasive, muscle-implanted NIRS in seals [25,26]. However, the absorption coefficient and reduced scattering coefficient for seal tissues are not yet published. Therefore, a DPF of six has been assumed for all measurements [58]. The PortaLite mini consisted of a sensor body, housed in an aluminium case with a removable O-ring sealed lid, and a sensor head. To waterproof the sensor head, the light diodes, photodiode receiver, and printed circuit boards (PCBs) of the sensor head were first fitted into optically opaque polyoxymethylene housings. The housings were filled with spectrally transparent epoxy (EPO-TEK 301, Epoxy Technology, Billerica, MA, United States). To ensure that epoxy encapsulated the electronics but did not cover the optical window on the optodes, the sensor head was cradled in a custom-built silicone mould. This allowed the internal components to be filled and waterproofed by epoxy but ensured the external surface and LEDs remained exposed. Once the housings were filled with epoxy, an optically opaque lid was placed onto the housing and fixed with two screws. Finally, the exterior of the housings and interoptode were potted in optically transparent epoxy for additional robustness and waterproofing. This solution for waterproofing ensured that light intensity was not impacted by the waterproofing process. Distance between light sources and detector were 28 mm (850 nm and 751 nm optode), 33 mm (852 nm and 751 nm optode), and 38 mm (851 nm and 752 nm optode), allowing optical penetration to three different depths (larger spacing between the optode and receiver allows deeper optical penetration; related to Fig 1). The response time of the PortaSeal was ≤0.1 s.

#### Seal diving trials

Diving trials were conducted with four temporarily captive harbour seals in a purpose-built foraging pool at the Sea Mammal Research Unit, University of St Andrews [52]. The PortaSeal was attached to a seal prior to each recording session during brief manual restraint of <1 min. The sensor body was attached to a baseplate superglued (Loctite 4861, Henkel, Winsford, United Kingdom) to the fur at the base of the skull. The sensor head was inserted into a photopolymer chassis superglued (Loctite 4861, Henkel, Winsford, UK) to the fur on either an animal’s head or shoulders and covered with an aluminium plate to maintain contact between the optodes and skin. An area of fur equal to the inner area of the chassis was trimmed to the level of the skin using rabbit-nosed clippers. The seal was then held for 5–15 min in a holding pen with access to ambient air to gain prediving NIRS measures.

A diving session started by opening an underwater gate in the pen, allowing access to the simulated foraging setup in a covered 32 × 6 × 2.4 m pool. The seal was then free to make repeated swims between a breathing chamber and a feeding station [52] (S3 Fig). An arrangement of lanes allowed the feeding station to be set at 58 m swimming distance from the breathing chamber. The seal could only surface in the breathing chamber but was allowed to dive and surface ad libitum. An experiment ended once the seal had taken a predetermined (specific for an individual’s body mass) amount of fish at the feeding station. The seal then remained in the holding pen for 10–30 min to acquire postdiving spectrographic measurements. The PortaSeal was then removed and data downloaded to a PC.

#### NIRS system location

For cerebral measurements, the sensor head was located over the parietal lobe of the right hemisphere, approximately 2 cm from the longitudinal cerebral fissure. Location of the parietal lobe was determined by locating three anatomical features—the sagittal crest and the posterior ridge of the parietal and the caudal ridge of the eye socket. For blubber measurements, the sensor head was located on the ventral surface of the shoulder region. Skin, blubber, and skull depths at each sensor head location were measured, prior to photopolymer chassis attachment, with an ultrasound scanner (CS-3000, Diagnostic Sonar, Livingstone, UK).

#### Tissue depth

Depth from the skin surface to the brain was 8 mm in all animals (including 2 mm skin depth and 1 mm skull depth). Depth of blubber on shoulder region ranged from 17 to 20 mm (including 2 mm skin depth).

### Quantification and statistical analysis

Dives were divided into four behavioural states: descent (i.e., swimming to the feeder), at the feeder, ascent (swimming to the breathing chamber), and PDSI. NIRS data were postprocessed using Oxysoft (Artinis Medical Systems, Einsteinweg, the Netherlands), and a 5 s moving Gaussian filter was applied to smooth the data and remove movement artefacts. The start of the first dive of each recording session was taken as baseline (0 μ.mol.L^−1^). Mean and standard deviation values were generated using R. Data were deposited in the Dryad repository: https://datadryad.org/review?doi=doi:10.5061/dryad.k67cg66.

## Supporting information

S1 Fig Example traces of blubber blood volume and haemoglobin oxygen dynamics from each of three optode channels during a sequence of dives from two animals: (A) Thor and (B) Soren. Green lines represent blood volume [tHb], and black lines represent haemoglobin oxygenation [Hb_diff_]. Dot and dashed lines represent measurements from the shallowest channel (28 mm), dashed lines represent measurements from the middle channel (33 mm), and solid lines represent measurements from the deepest channel (38 mm). [Hb_diff_], difference in the concentration of oxy- and deoxyhaemoglobin; [tHb], concentration of total haemoglobin.
(TIF)

S2 Fig Example trace of cerebral blood volume and haemoglobin oxygen dynamics from each of three optode channels during a sequence of dives from a second animal (Vebjörn). Green lines represent blood volume [tHb], and black lines represent haemoglobin oxygenation [Hb_diff_]. Dot and dashed lines represent measurements from the shallowest channel (28 mm), dashed lines represent measurements from the middle channel (33 mm), and solid lines represent measurements from the deepest channel (38 mm). [Hb_diff_], difference in the concentration of oxy- and deoxyhaemoglobin; [tHb], concentration of total haemoglobin.
(TIFF)

S3 Fig (A) Diagram of the experimental pool setup showing the location of experimental equipment and of a single lane divider required to provide a transit distance of 58 m between breathing chamber and feeding station. (B) Image of the breathing chamber with a seal during an interdive interval. (C) Image of a seal at the feeder where a belt provides a continual delivery of fish to a stationary seal.
(TIF)

S1 Movie Visualisation of the blood volume [tHb] and oxygenation [Hb_diff_] dynamics across a single dive. Gaussian-filtered (FWHM: 5 s) tHb and Hb_diff_ traces are projected to the MNI-ICBM-152 (2009) template of a human brain using Oxysoft (v. 3.2.24, Artinis Medical Systems, Einsteinweg, the Netherlands). The NIRS-trace replay speed is temporally synchronised with underwater footage. The position of transmitters and receivers are approximates, and the colour scale is between −4 (red) and 4 μMol (blue) change. The displayed data are of a 4 min dive condensed into a 60 s video. FWHM, full width at half maximum; [Hb_diff_], difference in the concentration of oxy- and deoxyhaemoglobin; [tHb], concentration of total haemoglobin.
(MP4)

S1 Table Summary of the data analysed in this study for each seal by tissue type and depth from the skin surface to muscle in blubber tissue and to cerebral tissue in brain measurements as measured by ultrasound scanning. (DOCX)

S1 Text Supplementary discussion of NIRS signal interpretation and estimation of CBV. CBV, cerebral blood volume; NIRS, near-infrared spectroscopy.
(DOCX)

## Abbreviations

BBV: blubber blood volume
BOLD: blood oxygen level–dependent
CBV: cerebral blood volume
CO: cardiac output
CW: continuous wave
DPF: differential pathlength factor
fNIRS: functional NIRS
Hb: haemoglobin
[Hb_diff_]: difference in the concentration of oxy- and deoxyhaemoglobin
[HHb]: concentration of deoxyhaemoglobin
HR: heart rate
NIRS: near-infrared spectroscopy
[O_2_Hb]: concentration of oxyhaemoglobin
PDSI: postdive surface interval
SRS: spatially resolved spectroscopy
[tHb]: concentration of total haemoglobin
TSI: tissue saturation index
