# Supplementary figures and images for: Shining new light on mammalian diving physiology using wearable near-infrared spectroscopy
Source: PLoS Biol. 2019 Jun 18;17(6):e3000306. doi: 10.1371/journal.pbio.3000306 (PMC6581238; doi:10.1371/journal.pbio.3000306)

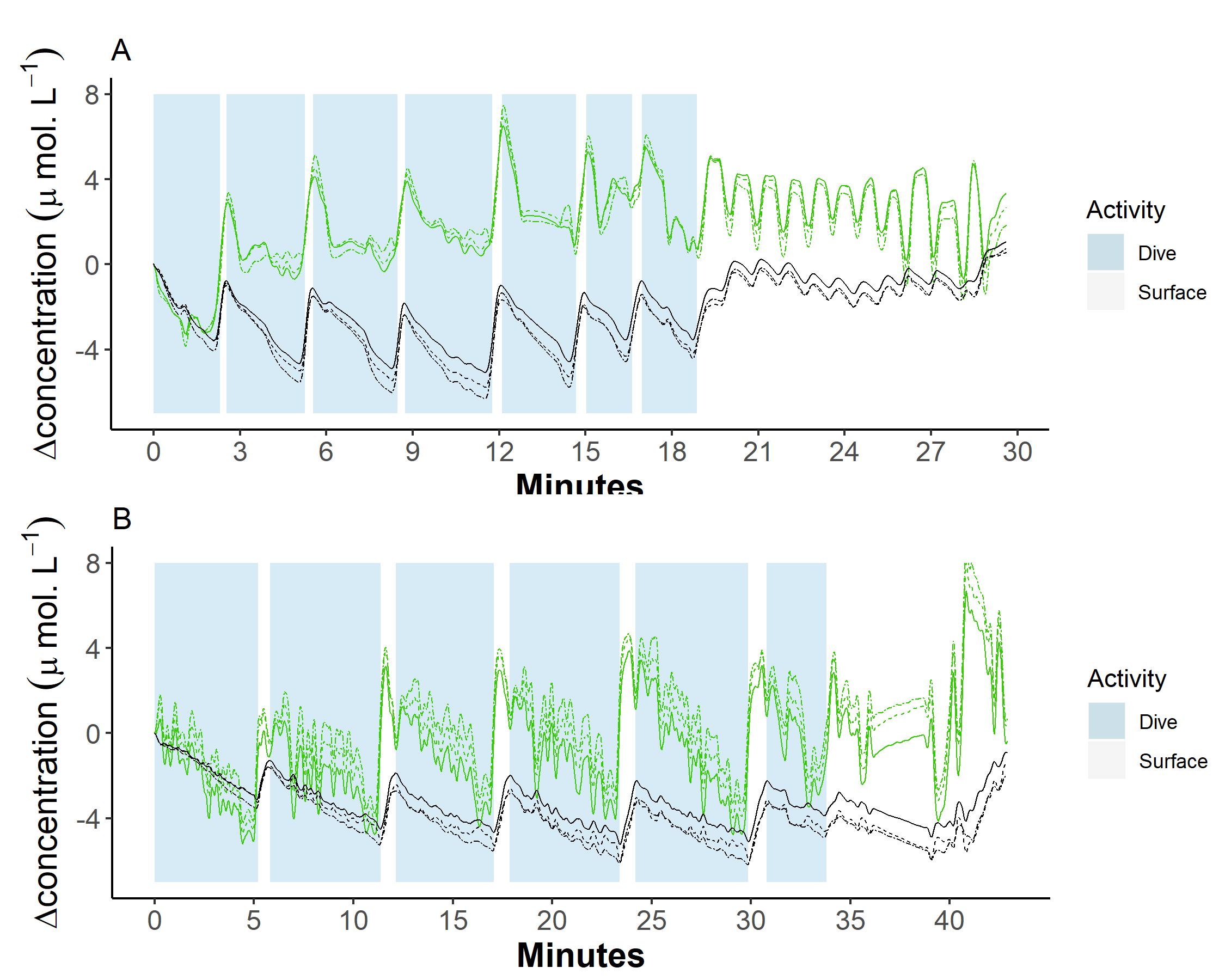

Supplement: S1 Fig — Green lines represent blood volume [tHb], and black lines represent haemoglobin oxygenation [Hbdiff]. Dot and dashed lines represent measurements from the shallowest channel (28 mm), dashed lines represent measurements from the middle channel (33 mm), and solid lines represent measurements from the deepest channel (38 mm). [Hbdiff], difference in the concentration of oxy- and deoxyhaemoglobin; [tHb], concentration of total haemoglobin. (TIF) [file pbio.3000306.s001.tif]

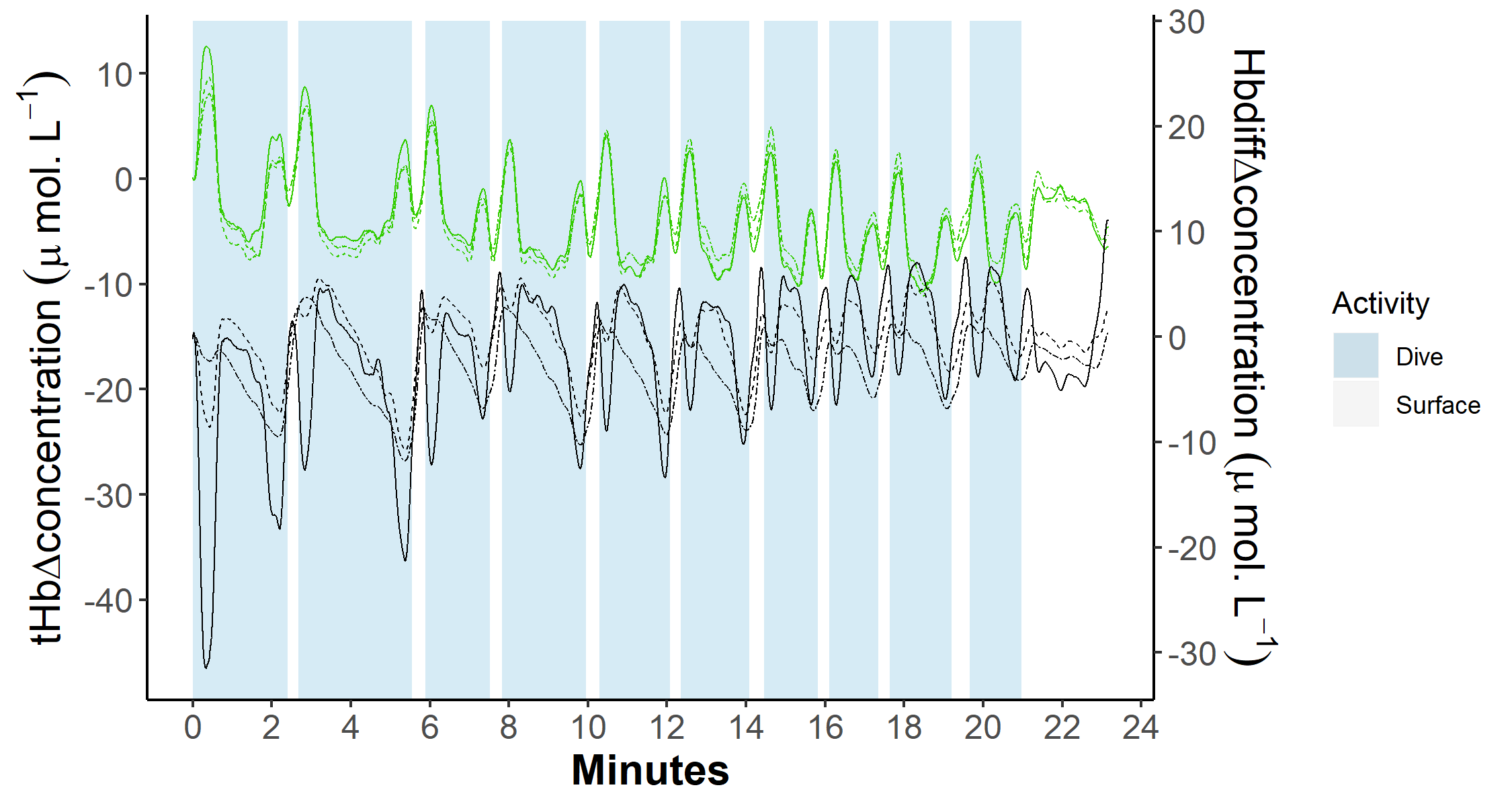

Supplement: S2 Fig — Green lines represent blood volume [tHb], and black lines represent haemoglobin oxygenation [Hbdiff]. Dot and dashed lines represent measurements from the shallowest channel (28 mm), dashed lines represent measurements from the middle channel (33 mm), and solid lines represent measurements from the deepest channel (38 mm). [Hbdiff], difference in the concentration of oxy- and deoxyhaemoglobin; [tHb], concentration of total haemoglobin. (TIFF) [file pbio.3000306.s002.tiff]

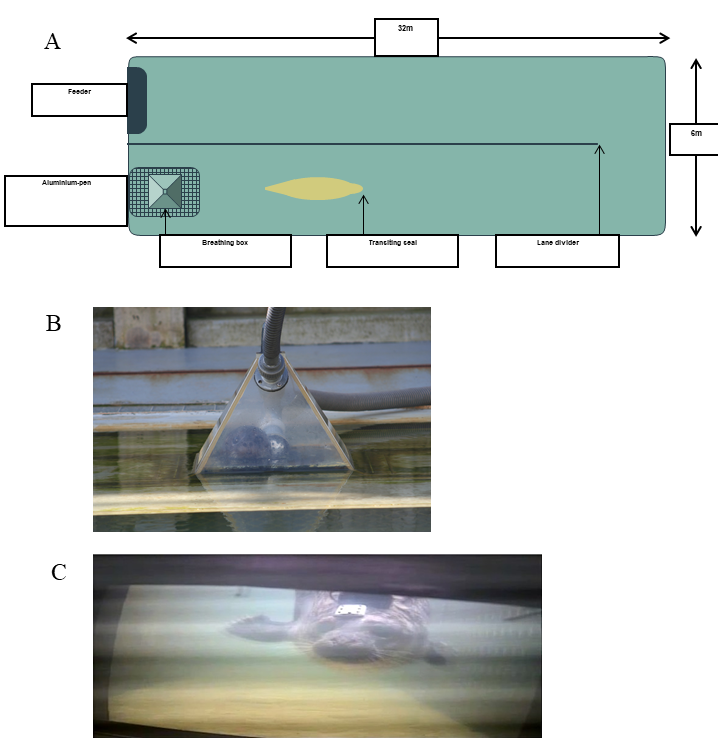

Supplement: S3 Fig — (A) Diagram of the experimental pool setup showing the location of experimental equipment and of a single lane divider required to provide a transit distance of 58 m between breathing chamber and feeding station. (B) Image of the breathing chamber with a seal during an interdive interval. (C) Image of a seal at the feeder where a belt provides a continual delivery of fish to a stationary seal. (TIF) [file pbio.3000306.s003.tif]
