# Supplementary material for: Shining new light on mammalian diving physiology using wearable near-infrared spectroscopy
Source: PLoS Biol. 2019 Jun 18;17(6):e3000306. doi: 10.1371/journal.pbio.3000306 (PMC6581238; doi:10.1371/journal.pbio.3000306)
Supplement: S1 Table — (DOCX) [file pbio.3000306.s005.docx]

| Animal | Blubber Trials  (dives) | Brain Trials (dives) | Depth to muscle layer (mm) | Depth to brain (mm) | Animal  Mass (kg) |
| --- | --- | --- | --- | --- | --- |
| Ulf | 4 (26) | 9 (54) | 17 | 8 | 43.6 |
| Søren | 6 (47) | 0 | 14 | NA | 43.0 |
| Thorston | 5 (28) | 0 | 13 | NA | 40.0 |
| Vebjørn | 0 | 3 (22) | NA | 8 | 37.2 |
