# Supplementary material for: Shining new light on mammalian diving physiology using wearable near-infrared spectroscopy
Source: PLoS Biol. 2019 Jun 18;17(6):e3000306. doi: 10.1371/journal.pbio.3000306 (PMC6581238; doi:10.1371/journal.pbio.3000306)
Supplement: S1 Text — CBV, cerebral blood volume; NIRS, near-infrared spectroscopy. (DOCX) [file pbio.3000306.s006.docx]

**Supplementary Discussion**

**Interpreting NIRS signal**

NIRS has previously been used in diving animals to measure muscle oxygenation [16,17]. In all cases, the light emitting diodes and receivers were inserted into the muscle tissue. Direct insertion into the muscle removes interference and contamination of the NIRS signal by other tissues. In the present study, both the light-emitting diodes and photodiode receiver were mounted superficially on the skin of seals. Importantly, the NIR light must pass through other tissues before interrogating the brain or blubber. In the case of cerebral measurements, for example, light must first traverse skin, a thin blubber layer and skull before reaching the brain. The light must then traverse these tissues again, before detection at the skin surface. It is therefore important to consider two questions: 1) Are the NIRS signals and dynamics representative of brain and blubber and; 2) What is the contribution of other tissues to the signals and dynamics?

Cerebral measurements

In human applications of NIRS the penetration depth of near-infrared (NIR) light into tissue is assumed to be approximately half the distance between the light sources and detector [19]. If this assumption was applied here for seals, then even the shortest separation distance used in the present study (28mm), would penetrate ~14mm, which would reach cerebral tissue, considering that distance from skin to brain (as measured using ultrasound) was 8 mm for both seals. Indeed, juvenile harbour seals provide an excellent model for non-invasive NIRS because the distance from skin to brain is only half that of adult humans (~17 mm), and, the skull is thin relative to humans (1 mm in the present study). Since the optical properties of seal tissues have not been measured, we use the observed NIRS data to assess whether or not our data represent the appropriate tissue.

The kinetics of [tHb] and [Hb_diff_] should be the same in all channels if the light from each optode-receiver separation distance had interrogated cerebral tissue. The kinetics of both [tHb] and [Hb_diff_] during diving were the same in each channel (Fig 4, S2 Fig), resulting in a TSI signal with kinetics matching the dynamics of [Hb_diff_] from all channels (Fig 4, Fig 5) suggesting that the NIRS data presented here represent cerebral changes in blood volume and oxygenation.

While the dynamics of each optode-receiver separation showed the same kinetics, the magnitude of change differed between separation distances. the magnitude of change in [Hb_diff_] increased with optode-receiver separation distance (Fig 5; S2 Fig). For example, during the first dive (Fig 4), secondary reoxygenation results in a clear increase in [Hb_diff_] in the 38mm (deepest) channel. Secondary reoxygenation is less pronounced in the middle channel (33mm) and in the shortest/shallowest channel (28mm) secondary reoxygenation does not appear, and [Hb_diff_] remains constant. Diminishing cerebral signal in the shallower channels may be a result of increased contribution of tissues above the brain to the NIR signal. In the present study, the contribution of non-brain tissues is difficult to quantify as all the optode-receiver channels have ultimately interrogated cerebral tissue. Inclusion of a short optode-receiver separation distance, e.g. 1cm, would provide estimates for skin and blubber without cerebral signal. That signal could then be removed from each of the three current channels using a short-separation regression [59] to provide less contaminated cerebral NIRS signal.

Blubber measurements

Assumption that penetration depth of NIR light is approximately half the distance between the light sources and detector, even the longest optode-receiver separation (38mm) signals would have interrogated skin and blubber only. The light would not have penetrated to reach the underlying muscle for two animals with blubber depths of 20mm. For one animal, with blubber depth of 17mm, light from the 38mm optode channel may have penetrated to muscle underlying the blubber. The fact that kinetics of [tHb] and [Hb_diff_] were the same for each optode-receiver separation (Fig 5 & S3) suggests that either no muscle penetration occurred or any resulting signal was insufficient to influence the patterns. Furthermore, given the high scattering coefficient of adipose tissue, at even the maximum optode-receiver separation distance of 38mm, the sensitivity of NIRS to muscle O_2_ changes under 17mm of skin and adipose tissue is likely to be poor [60].

While the dynamics of each optode-receiver separation showed the same kinetics, the magnitude of change differed between separations. For example, the magnitude of change in [Hb_diff_] decreased with greater optode-receiver separation distance. Fig 3 shows that while the magnitude of change in haemoglobin oxygenation is similar between the 28mm and 33mm optode-receiver channels, magnitude of deoxygenation is lower in the 38mm channel (Fig 3, S1 Fig). This pattern indicates that haemoglobin changes in shallow tissue, such as the skin or shallow blubber, have a greater contribution to the dynamics reported here for blubber. Similar to cerebral measurements, skin ‘contamination’ could be removed by use of a shorter optode-receiver separation [59].

Comparing brain and blubber measurements

The kinetics of [tHb], [Hb_diff_] and TSI at the same sites were common between animals. However, differences existed between cerebral and blubber measurements, which demonstrate that the results presented accurately reflect haemodynamics of different tissues. If the NIRS signals presented here were not from different tissues, for instance if NIR light had only interrogated skin and blubber, then the brain and blubber kinetics would not differ.

Theoretical Calculation of CBV Change

Assuming that blood volume per litre of brain tissue in seals is equal that of humans (50 ml of blood per litre [61]) and taking 0.2g of Hb per ml of blood for a harbour seal [62], this equates to 10g of Hb per litre of brain tissue. The four tetramers of Hb have a total weight of 64000 g.mol^-1^. 10g Hb ÷ 64000 g.mol^-1^ = 156 μ.mol Hb per litre of brain tissue. Therefore a ~5 .mol Hb per litre of brain tissue change in CBV during the second half of each surface interval results in a 3.2% increase in CBV from pre-diving baseline levels. As CBV is not known for seals the human value is used here for theoretical purposes. As the brain of seals has a greater capillary density [63] and the ~5 .mol Hb per litre of brain tissue includes some contribution of tissues dorsal of the brain, the value is reported as ≤3.2%.

**Additional References**

59. Yücel M.A., Aasted C.M., Petkov M.P., Borsook D., Boas D.A., and Becerra L. Specificity of hemodynamic brain responses to painful stimuli: A functional near-infrared spectroscopy study. Sci. Rep. 2015; 5, 9469.

60. Matsushita K., Homma S., and Okada E. Influence of adipose tissue on muscle oxygenation measurement with NIRS instrument. Proc. SPIE Int. Soc. Opt. Eng. 1998; 3194: 159–165.

61. Sakai F., Nakazawa K., Tazaki Y., Ishii K., Hino H., Igarashi K., and Kanda T. Regional cerebral blood volume and haematocrit measured in normal human volunteers by single-photon emission computed tomography. J. Cereb. Blood Flow Metab. 1985;5(2): 207-213.

62. Clark C.A., Burns J.M., Schreer J., Hammill M.O. A longitudinal and cross-sectional analysis of total body oxygen store development in nursing harbour seals (Phoca vitulina). J. Comp. Physiol. B. 2007;177(2): 217 – 227.

63. Kerem D., and Elsner R. Cerebral tolerance to asphyxial hypoxia in the harbor seal. Resp. Physiol. 1973; 19(2): 188-200.
